# The evolution of postmortem investigation: a historical perspective on autopsy's decline and imaging's role in its revival

**DOI:** 10.3389/fradi.2025.1565012

**Published:** 2025-04-14

**Authors:** Nadia Solomon, Dominic Gascho, Natalie L. Adolphi, Laura Filograna, Harold Sanchez, James R. Gill, Jamie Elifritz

**Affiliations:** ^1^ Department of Radiology and Biomedical Imaging, School of Medicine, Yale University, New Haven, CT, United States; ^2^ Investigative Medicine Program, Yale Graduate School of Arts and Sciences, Yale University, New Haven, CT, United States; ^3^ Forensic Pathology, New York City Office of Chief Medical Examiner, New York, NY, United States; ^4^ Institute of Forensic Medicine, University of Zurich, Zurich, Switzerland; ^5^ Center for Forensic Imaging, Office of the Medical Investigator, University of New Mexico, Albuquerque, NM, United States; ^6^ Integrated Care Processes Department, UOC of Diagnostic Imaging, University of Rome, Rome, Italy; ^7^ Department of Pathology, School of Medicine, Yale University, New Haven, CT, United States; ^8^ Forensic Pathology, Connecticut Office of the Chief Medical Examiner, Farmington, CT, United States; ^9^ The Forensic Radiology Group, Anderson, SC, United States

**Keywords:** postmortem imaging, forensic medicine, autopsy, postmortem computed tomography (PMCT), pathology, radiology, virtual autopsy

## Abstract

Autopsy is generally regarded as the gold standard for cause of death determination, the most accurate contributor to mortality data. Despite this, autopsy rates have substantially declined, and death certificates are more frequently completed by clinicians. Substantial discrepancies between clinician-presumed and autopsy-determined cause of death impact quality control in hospitals, accuracy of mortality data, and, subsequently, the applicability and effectiveness of public health efforts. This problem is compounded by wavering support for the practice of autopsy by accrediting bodies and academic bodies governing pathology specialty training. In forensic settings, critical workforce shortages combined with increased workloads further threaten sustainability of the practice. Postmortem imaging (PMI) can help mitigate these ongoing problems. Postmortem computed tomography can help clarify manner and cause of death in a variety of situations and has undeniable advantages, including cost reduction, the potential to review data, expedient reporting, archived unaltered enduring evidence (available for expert opinion, further review, demonstrative aids, and education), and (when feasible) adherence to cultural and religious objections to autopsy. Integration of radiology and pathology is driving a transformative shift in medicolegal death investigations, enabling innovative approaches that enhance diagnostic accuracy, expedite results, and improve public health outcomes. This synergy addresses declining autopsy rates, the forensic pathologist shortage, and the need for efficient diagnostic tools. By combining advanced imaging techniques with traditional pathology, this collaboration elevates the quality of examinations and advances public health, vital statistics, and compassionate care, positioning radiology and pathology as pivotal partners in shaping the future of death investigations.

## The autopsy: historical context and scientific contributions

“*Hic locus est ubi mors gaudet succurrere vitae.”* [This is the place where death delights to help the living.] – frequently attributed to anatomist Giovanni Morgagni (1, 2).

The earliest described systematic dissections of human bodies are thought to have been performed in the 3rd or 4th century BC by the Greek physician Herophilus, during a brief period when the idea of postmortem dissection seemed acceptable (3). Centuries later, Herophilus and colleagues were accused of performing vivisections on the living; but whether this is true may forever remain a mystery, as any written records are believed to have been destroyed in 391 AD by the fire that demolished the Library of Alexandria (3). For many centuries thereafter, historical records indicate that autopsy was largely opposed, among various reasons, for being immoral and potentially dangerous, both to the physical health of the living and, if performed carelessly or incorrectly, to the decedent's soul (3). During this time, the advancement of medical science was largely stifled due to negative attitudes towards human dissection, widespread acceptance of inaccurate anatomy and physiological concepts (e.g., due to adherence to Galen's teachings, which were mainly based on animal dissections), and a lack of scientific method or systematic approach to postmortem investigation (3, 4).

Even into the 17th century, historical works indicate that many prominent physicians believed autopsy to be useless (4). English physician Thomas Sydenham, for example, remarked that those who practiced autopsy did so “with how little success,” and perhaps he was not entirely incorrect: of the few who performed autopsies, even fewer seemed to understand the nature or importance of their findings (4). During medieval times, although professors performed cadaveric dissections for student audiences, anatomical observations differing from Galen's descriptions would be attributed to the individual, and not to species-dependent anatomical variations (3). It was, in fact, an artist, not a physician, whose transition from animal to human dissection led to several major discoveries: Leonardo da Vinci produced hundreds of drawings of human anatomy and various pathologies, and is posthumously credited as being the first to depict coronary artery anatomy and atherosclerosis (3). This work, however, was predominantly unpublished. It was not until the publication of *De Humani Corporis Fabrica*, an illustrated anatomy textbook based on human dissection written in the 16th century by Flemish physician Andreas Vesalius, that some of the anatomical misinformation would be corrected (3).

While Vesalius's text promoted the study of anatomy and suggested its clinical utility, it was only in the mid-1700s that autopsy's vital role in medicine began to be truly realized (5). By the 1800s and early 1900s, autopsy represented the greatest contributor to medical scientific discovery (4). Its rapidly growing popularity, however, also created a market for bodies, leading to grave-robbing, the Anatomy Act of 1832 (allowing for legal dissection of the unclaimed poor), and even a series of murders by the infamous William Burke and William Hare, who sold their victims’ corpses to a professor at the University of Edinburgh (3). Nevertheless, by 1900, the practice of autopsy carried such importance that Dr. Abraham Jacobi—who would become the president of the American Medical Association—felt it necessary to remind the 13th International Medical Congress in Paris that modern medicine is “not only diagnosis and autopsy, but the treatment and care of patients.” It was a widely shared sentiment at the time that the study of death could be used to benefit the living – a fact which remains true to this day. To this end, the autopsy provides incredible value, not only as the ground truth of medical diagnosis, but as a means by which physicians can improve their diagnostic skills and, subsequently, the quality of clinical care they provide. In fact, many prominent American medical scholars spent at least a year on the study of anatomic pathology (4). A true autopsy enthusiast, Dr. William Osler was notorious for following his patients at Johns Hopkins School of Medicine to autopsy and was known to perform many autopsies himself. Leading up to his own death, which was preceded by several months of illness, he remarked to a friend and colleague: “I’ve been watching this case for two months and I’m sorry I shall not see the postmortem.” Perhaps not surprisingly, the course of Dr. Osler's autopsy was dictated by his own strict instructions (4).

## Decline of the autopsy and its implications for modern medicine and forensic pathology

While autopsy with histopathology is regarded as the gold standard for determining cause of death, hospital autopsy rates have declined substantially since the 1950s – once performed in 50% of cases in the United States (US), rates decreased to 7.4% by 2020 (6, 7). In the United Kingdom (UK) and Wales, rates are now less than 1% (8, 9). With the emergence of the COVID-19 pandemic, concern for the potential risk of exposure to and infectivity of aerosolized virus during lung dissection resulted in the temporary cessation of autopsy at many hospitals worldwide (10). Although viewed at the time as a necessary protective measure, some researchers have since posited that the resultant absence of data on exact cause of death delayed understanding of disease pathophysiology and development of effective treatment strategies (11).

Irrespective of COVID, the decline in autopsy rates has occurred for a variety of reasons, including families declining autopsy, physicians not pursuing autopsy, fear of medicolegal consequences from missed diagnoses, and high cost, among others (9, 12–15). Select seminal events have been identified for their unfavorable effects on the appreciation and practice of autopsy. In 1971, the Joint Commission eliminated its requirement for a minimum number of autopsies to maintain hospital accreditation, implicitly reinforcing the perception that autopsies are not medically essential (16). By 1986, the Centers for Medicare & Medicaid Services ceased reimbursing autopsies, leading to funding cuts that compounded existing financial constraints and created significant, arguably insurmountable, barriers to sustaining the practice (16, 17). By 2020, the American Board of Pathology (ABPath), and subsequently the Accreditation Council for Graduate Medical Education, reduced the autopsy requirements for graduating pathology residents from 50 to 30 (10, 18). Some pathologists have even suggested removing autopsy from the residency training curriculum altogether, arguing that the time otherwise dedicated to autopsy training could be reallocated to training in newer disciplines and technologies (e.g., molecular genetics, informatics) (16). Those who have adopted this mindset for their training, however, may face difficulties in clinical practice: as noted by the Association of Pathology Chairs' Autopsy Working Group in their 2018 report, seasoned pathologists had already started lodging complaints with the ABPath that their new hires—purportedly recently certified in anatomic pathology—were unprepared and thus incapable of performing autopsies despite it being a requirement of their positions (16).

Given the variable attitudes toward autopsy (even among some pathologists), it is understandable that individuals without specialized training in pathology may have a limited understanding and appreciation of its significance. A deeper comprehension is essential to fully recognize its value. A study by Scarl et al. (2022) identified the belief that the cause of death was already known as the most common reason families declined autopsy (14). However, research shows that at least 25% of autopsies reveal clinically missed diagnoses related to the cause of death (19–22). Diagnostic discrepancies persist even when clinicians are “certain” of their diagnoses, with a 25% discrepancy rate rising to 54% when clinicians are “uncertain” (22, 23). A meta-analysis of 18 studies found discrepancy rates for cause of death ranging from 30% to 63%, with more than 20% of perioperative deaths potentially preventable with correct diagnosis and management (22).

These findings underscore the essential role of autopsy as a quality control measure in hospitals. Even in populations with consistent autopsy practices, such as stillbirths and infant deaths, autopsy altered the cause of death determination in 9%–10% of cases, significantly influencing genetic counseling and mortality statistics (24). With death certificates serving as the foundation of mortality data, low autopsy rates exacerbate clinicopathologic discrepancies, undermining the accuracy of mortality statistics. This, in turn, affects public health surveillance and healthcare planning, highlighting the critical need to preserve and prioritize autopsy practice (22, 25).

Within the field of pathology, the decreased support for the practice of autopsy by both hospitals and organizations regulating pathology training likely has the most detrimental effect on the subspecialty of forensic pathology, a unique discipline that combines medicine, science, and law, and relies on autopsy as its primary investigative technique (1). Currently, the forensic setting faces a critical international shortage in the forensic pathology workforce: there are only approximately 750 full-time, board-certified forensic pathologists practicing in the United Sates, and this is estimated to be half (if not less) the number needed (1, 26). Comparable shortages of forensic pathologists are noted globally. With a growing population of aging (and subsequently retiring) forensic pathologists, the numbers continue to dwindle, and are not off-set by newly-certified trainees (26).

In the United States, only around 40 pathologists achieve board certification in forensic pathology annually, highlighting the field's status as a highly specialized and limited resource with a lengthy developmental timeline. This certification requires a minimum of three years of residency in anatomic pathology (or four years in combined anatomic-clinical pathology), followed by an additional fellowship year in forensic pathology. Many forensic pathologists further extend their training with subspecialties such as neuropathology, cardiac pathology, or pediatric pathology, as well as related disciplines like anthropology or toxicology, underscoring the significant time and expertise required to cultivate a qualified forensic pathologist (1, 26). Recent events have only exacerbated the workforce shortage, including high death tolls related to the opioid epidemic and the COVID-19 pandemic (27, 28). Historically, compensation has been a significant deterrent to pursuing forensic pathology. For many years, forensic pathologists, including Chief or Deputy Chief Medical Examiners, earned only 50%–75% of what anatomical pathologists in hospital settings made. However, recent efforts have been aimed at narrowing this pay gap to make the field more competitive (17).

## A brief introduction to forensic imaging and the virtual autopsy

It did not take long after Roentgen's discovery of x-rays in 1895 for scientists to begin to explore its application to medicine (29). In fact, the influential role imaging would play in both clinical and forensic medicine seemed intuitive not only to physicians and scientists, but to the public, with the January 6, 1895, installment of the New York Sun prematurely, yet predictively, reporting that Dr. Roentgen was “already using his discovery to photograph broken limbs and bullets in human bodies” (30). Before being applied to humans, however, the technology was applied to a different animal: Professor A. W. Wright of Yale University acquired a radiograph of a rabbit, documenting the presence and location and facilitating the extraction of several small round projectiles, thereby establishing the animal's cause of death (30). By Christmas Eve of 1895, the first court case in North America to utilize imaging as evidence commenced; this Canadian case featured an x-ray plate of the leg of a shooting victim with a retained bullet and resulted in a conviction for attempted murder. Within the year, imaging evidence would be introduced in international courts in both criminal and civil cases, although its acceptance was not immediate nor universal. The dangers of radiation would also soon be realized, with radiation-mediated damage to one man's ankle in late 1896 leading to amputation and a successful malpractice lawsuit by the plaintiff (30).

As radiology took hold in medicine and in the courtroom, it would also be applied in postmortem investigations, including in famous cases like the assassination of President John F. Kennedy (although apparently causing more controversy than clarity in this case) and even in the identification of the remains of Adolf Hitler (a finding kept secret by the Russians for over two decades) (30).

In the 1970s, computed tomography (CT) revolutionized clinical medicine. Like autopsy, CT is a powerful diagnostic tool for visualizing the internal structures of the body. In contradistinction, CT offers a non-invasive window, enabling examination without physical alteration. Clinical adoption was quickly followed by its application to postmortem investigations. Dr Byron Gilliam “Gil” Brogdon, internationally known and recognized as the Godfather of Forensic Radiology, published the seminal reference for postmortem imaging (PMI) in 1998, *Forensic Radiology*. His contribution to the field includes his numerous international collaborations and mentorship of budding forensic pathologists and radiologists into this new subspecialized field. In 2000, the Institute of Legal Medicine at the University of Bern in Switzerland launched the Virtopsy® Project, with the mission to develop standardized protocols for integrating PMI techniques into forensic investigations (29, 31, 32). Despite its rich and intriguing history, forensic radiology has only recently emerged more prominently as a recognized subspecialty, particularly in the past few decades and following the global recognition of the Virtopsy® Project (32).

The term *virtopsy* is derived from the words “virtual”—from the Latin *virtus*, meaning “virtue”—and “autopsy”—from the Greek *autos* and *opsomei*, meaning “to see for oneself” (33). Intentional exclusion of the root *autos* to form the term *virtopsy* by Virtopsy® Project founding members represented an effort to suggest the elimination of subjectivity; although it is important to note that, while the stand-alone imaging data may itself be objective, the necessity of its interpretation by a radiologist (or forensic pathologist) prevents subjectivity from being eliminated entirely (33).

## Benefits and limitations of postmortem imaging

Despite the numerous benefits of autopsy, it has important limitations. Relying on a combination of dissection, descriptions of findings, and diagrams/photographs, the process—like any other process performed by humans—is subject to human error (34). Similarly, histology is limited by interpretation and sampling bias. With that in mind, perhaps the biggest limitation of autopsy is the fact that undetected or undocumented findings are irrevocably lost due to the process of autopsy, decomposition, and/or cremation (34). Incorporation of the virtual autopsy in postmortem investigation can help reduce the impact of these limitations.

### Benefits of postmortem imaging with comparison to conventional autopsy

A major benefit of virtual autopsy is that, unlike autopsy specimens, imaging data can be stored indefinitely and thus can be reviewed at any time (32, 33). Furthermore, when imaging is acquired prior to autopsy (a standard practice in centers that perform virtual autopsy), the image data effectively reflects the untouched, “zero-status” state of the decedent (32). Despite the subjectivity encountered with radiologic interpretation, the availability of the data facilitates sharing and a second opinion can be obtained (33).

**Figure 1:**
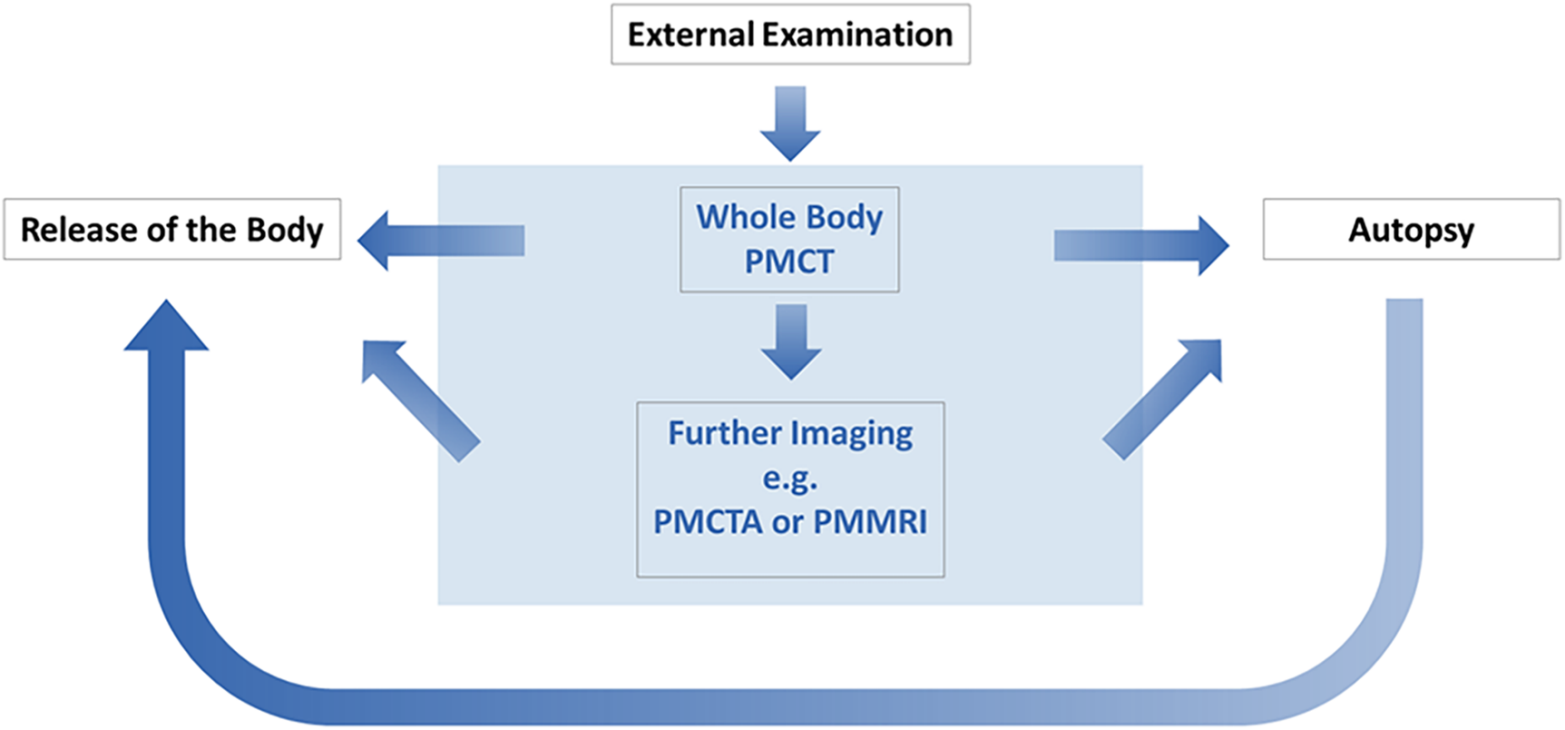
Schematic illustrating the integration of postmortem imaging at forensic centers for the purpose of autopsy triage.

PMI provides data on the circumstances and conditions leading to death and can verify or refute initial theories or clinical diagnoses even prior to performance of an autopsy (32). PMI, most commonly whole-body postmortem computed tomography (PMCT), has been adopted at several forensic centers to enhance and streamline death investigations. PMI serves multiple roles, including triaging decedents for external examination or autopsy (Figure 1) (35–37). It assists in identifying signs of external influence or foul play, aiding in the determination of the manner of death (how a person died, e.g., natural, accident, suicide, homicide, undetermined) and triggering further investigation with autopsy when warranted. Additionally, PMCT can be used to plan an autopsy by identifying areas of interest, synergize with autopsy findings to provide a more comprehensive understanding of the cause of death, or, under specific conditions, even supplant the need for an autopsy entirely (32, 38).

Due to short acquisition times, PMCT can be easily performed in the time frame between decedent arrival and autopsy commencement, and the practice allows for conservation of valuable time and resources in cases where imaging permits autopsy to be either targeted or forgone (32, 33). In the setting of critical workforce shortages in forensic pathology, these applications will likely play an increasingly influential role in supporting forensic pathologists and sustaining the field. However, despite its widespread acceptance (particularly in forensic settings) and rapid expansion of its applications, PMI remains underutilized, most notably in the United States (8, 9, 32).

**Figure 2:**
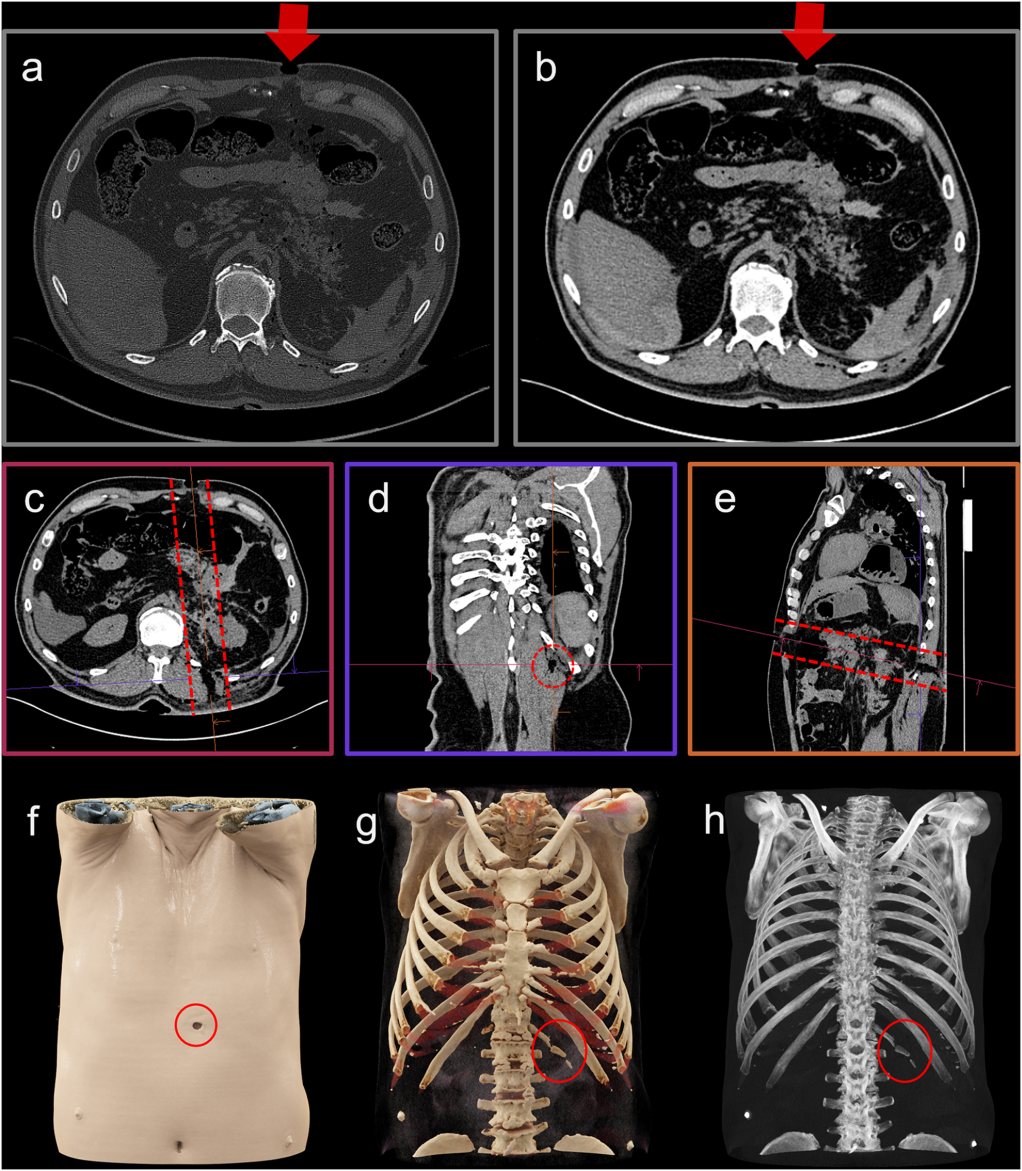
Case example depicting an abdominal gunshot wound (51). The axial slices with a hard kernel and bone window **(a)** and a soft kernel with soft tissue window **(b)** represent the two standard CT data visualizations. In these images, which display slices perpendicular to the scan axis, only the entry wound is visible (a and b, arrow). One advantage of virtual autopsy using CT is its ability to provide three-dimensional data, and through multi-planar reformations **(c–e)**, the orientation of key findings—such as the bullet trajectory [**(c–e)**, dashed markings]—can be adjusted accordingly. Three-dimensional visualizations, including volume rendering **(f)**, cinematic rendering **(g)**, and maximum-intensity projection **(h)**, offer impressive depictions that enhance understanding, even for non-radiologists. For example, the gunshot wound at the skin surface is clearly visible [**(f)**, red circle], as is a fracture of the 13th rib [**(g,h**), red circle]. Different visualization techniques can be selected depending on the specific findings; but whether those findings are recognized depends on the observer's training and experience.

Research into the diagnostic capabilities of whole-body PMCT is both promising and ongoing. In certain situations, PMCT has demonstrated superiority over traditional autopsy. For instance, it excels at identifying abnormal accumulations of air, such as pneumothorax and is highly effective at detecting and precisely localizing foreign bodies (32, 39). Additionally, PMCT's ability to differentiate between various metal components provides significant value in ballistic investigations, offering detailed insights that enhance forensic analyses (Figure 2) (40, 41). PMCT is more sensitive for skeletal injuries and injury patterns (which is particularly helpful in cases with major trauma) (32, 33). PMCT can be particularly helpful for diagnosing base of skull and cervical vertebral injury as autopsy dissection of these areas can be challenging and time consuming.

### Limitations of postmortem imaging and ongoing investigations to address them

Despite its numerous benefits, PMCT remains less effective than autopsy in depicting soft tissue and organ injuries, precisely locating vascular injuries, identifying various vascular pathologies, and assessing superficial injuries (32, 42). This limitation is further supported by the clinical literature that has demonstrated, as noted above, that at least 25% of autopsies reveal clinically missed diagnoses related to the cause of death despite the widespread use of clinical radiological imaging.

Applying advanced imaging techniques may help overcome some of these weaknesses. For example, investigations demonstrate that postmortem CT angiography (PMCTA) could vastly improve the characterization of vascular pathology, including vascular diseases and injuries and their sequelae; this area of research is currently garnering intense interest (8, 43, 44). In this technique, a contrast agent mixture is introduced into the vascular system via an access port to fill the vascular tree, and a CT scan is subsequently performed. PMCT-guided biopsy can be used to obtain samples for histological analysis or microbial testing, particularly in cases when there is objection to autopsy or risks associated with its performance (32, 45). Advanced CT imaging techniques such as high-resolution micro-CT (μCT, allowing for evaluation of tissue microstructure) can be used on excised tissue or bone samples, as well as on fetuses. Spectral CT technologies facilitating material differentiation—such as dual-energy CT (DECT) and photon-counting detector CT (PCD-CT)—are also anticipated to expand the diagnostic capacity of forensic and PMI investigations, although these techniques have thus far been applied predominantly in research settings (32). Postmortem magnetic resonance imaging (PMMRI) is also being explored due to its ability to provide superior soft tissue discrimination and fine details compared to CT; but its adoption remains limited, as the equipment is expensive, running the machine and developing protocols requires specialized training (including safety training), and the complicated technology is more difficult to adapt to postmortem environments (32).

## Current practices and the importance of careful integration

At the time of this manuscript, use of PMI is more often seen in forensic compared to clinical settings, and there remains widespread variability in its adoption and everyday use, both geographically and across institutions (32, 46). In the United States, PMI remains vastly underutilized in both clinical and forensic settings, especially compared to parts of Europe and Japan. There is also substantial variation in the type of PMI used (e.g., radiography, CT, or MRI), the role PMI plays in postmortem investigations (e.g., as a triage tool for full or limited autopsy or isolated external examination, or as a complementary or supplementary examination performed alongside autopsy), who performs the examination and acquires the images (e.g., imaging technologists, mortuary technicians, or physicians), and who performs the imaging interpretation (e.g., pathologists, radiologists, other physicians, or non-physicians) (36, 46). There is also variation in the extent of specific training in PMI interpretation received by those performing this task, as well as in how PMI interpretations are formally reported (36, 46). Cost of acquiring and maintaining the machinery and performing the examinations is another complex, geographically and institutionally dependent issue. Looking ahead, the establishment of international standards for PMI protocols, training, and reporting could help mitigate these disparities and promote more consistent integration of PMI into forensic and clinical workflows worldwide. As the global authority on forensic imaging, the International Society of Forensic Radiology and Imaging (ISFRI) develops and publishes evidence-based standards to ensure the accuracy, reliability, and legal admissibility of PMI, serving as the definitive reference for best practices (47–49).

The integration of new imaging technologies into postmortem investigations offers significant potential but must be applied judiciously to avoid the pitfalls seen in clinical medicine, where over-reliance on imaging has diminished essential investigative techniques such as physical examination (50). While imaging can aid in determining the cause of death and improve efficiency, appropriate and responsible use involves proper case selection, recognition of known pitfalls, and realistic expectations. Complete replacement of autopsy would compromise mortality data accuracy (43). Imaging can support forensic practices by preserving objective data, reducing strain on forensic pathologists, and mitigating the effects of declining autopsy rates on public health, medical research, and vital statistics.

When used appropriately, PMI serves as a valuable complement to autopsy, fostering collaboration between radiology and pathology to ensure that each case is approached with the most effective and appropriate tools. Rather than inadvertently reducing autopsy rates, PMI can enhance forensic investigations by (a) facilitating accurate diagnoses in cases not initially intended for autopsy, and (b) reducing the risk of missed findings by identifying occult injuries that might otherwise go undetected during external examination. This approach ensures that autopsies are reserved for cases where they are truly necessary, leading to a meaningful increase in autopsy rates when warranted and fostering a more effective and high-quality service to our communities.

## Conclusions

Over the last few centuries, autopsy has arguably provided some of the most significant contributions to modern medicine via innumerable discoveries in human anatomy and pathology; yet appreciation of these contributions is being progressively lost, reflected by declining autopsy rates witnessed over the past several decades. With research clearly demonstrating the pitfalls of relying on clinical diagnosis alone, the phenomenon of the declining autopsy represents a substantial threat to clinical medicine, public health, and forensic investigations, starting at least from the level of residency training, if not before.

Although it has not demonstrated the capacity to replace autopsy, PMI represents a partial yet extremely viable solution to several obstacles faced by the forensic pathology subspecialty, and to many more widespread scientific, educational, and public health problems posed by decreasing autopsy rates. With continued research, validation, and utilization, imaging has the potential to help alleviate some of the strain on the forensic pathology workforce; and (to a degree) offset the loss of data associated with declining autopsy practice, preserving the accuracy of public health data, the quality of clinical medical care, and the veracity of legal practice (36).

## Data Availability

The original contributions presented in the study are included in the article/Supplementary Material, further inquiries can be directed to the corresponding author.
